# A new species of *Gryllotalpa* (Orthoptera, Gryllotalpidae) from northwestern China, with notes on its mitochondrial genome

**DOI:** 10.3897/zookeys.1291.203191

**Published:** 2026-09-02

**Authors:** Ya-Rong Gu, Xin-Lan Deng, Ling Zhu, Yan Ma, Ying Miao

**Affiliations:** 1 School of Agriculture, Ningxia University, Yinchuan 750021, China School of Agriculture, Ningxia University Yinchuan China; 2 Bureau of Agriculture and Rural Affairs of Zhuxi County, Shiyan 442300, China Bureau of Agriculture and Rural Affairs of Zhuxi County Shiyan China

**Keywords:** Gene rearrangement, identification key, mitogenome, mole cricket, morphology, new species

## Abstract

A new species of mole cricket, *Gryllotalpa xinjiangica* Gu & Miao, **sp. nov**., is described from Xinjiang, northwestern China, based on morphological characters and molecular data. The new species belongs to the *G. gryllotalpa* species complex and represents the second confirmed species of this complex known from East Asia. It can be distinguished from related species by the absence of inner subapical spurs on the hind tibiae, tegminal venation, and the structure of the male phallic complex. The complete mitochondrial genome of *G. xinjiangica* Gu & Miao, **sp. nov**. was sequenced, revealing a novel tRNA gene rearrangement (*trnE-trnN-trnS1*). Phylogenetic analyses based on the mitochondrial *cox1* gene support the distinctiveness of the new species and recover it as sister to the *G. gryllotalpa* + *G. vineae* clade. An identification key to the known Chinese species of Gryllotalpidae is provided.

## Introduction

The family Gryllotalpidae (Orthoptera) comprises a group of specialized fossorial insects characterized by modified forelegs adapted for digging, enlarged pronota, and short antennae (Cadena-Castañeda 2015a). The family currently includes more than 100 described species in eight extant genera (Cigliano et al. 2026). Among these genera, *Gryllotalpa* is the most speciose and widely distributed, and is the only genus of Gryllotalpidae recorded from China. Species of *Gryllotalpa* are diagnosed by fore tibiae bearing four dactyls, slit-shaped tympanal openings, a basal femoral spur on the fore femur, and tegminal venation extending laterally toward the wing tips (Ma and Zhang 2010). To date, 76 species of *Gryllotalpa* have been described worldwide, with 12 species known from China (He 2018; Wang et al. 2024; Cigliano et al. 2026).

The *G. gryllotalpa* species complex is widely distributed throughout Europe and western to northern Asia and currently comprises 15 described species: *G. cossyrensis* Baccetti & Capra, 1978, *G. gryllotalpa* (Linnaeus, 1758), *G. isfahan* Ingrisch, Nikouei & Hatami, 2006, *G. krimbasi* Baccetti, 1992, *G. marismortui* Broza, Blondheim & Nevo, 1998, *G. octodecim* Baccetti & Capra, 1978, *G. quindecim* Baccetti & Capra, 1978, *G. sedecim* Baccetti & Capra, 1978, *G. septemdecimchromosomica* Ortiz, 1958, *G. stepposa* Zhantiev, 1991, *G. tali* Broza, Blondheim & Nevo, 1998, *G. unispina* Saussure, 1874, *G. viginti* Baccetti & Capra, 1978, *G. vigintiunum* Baccetti, 1991, *G. vineae* Bennet-Clark, 1970. However, only *G. unispina* has been recorded from China (Cai and Niu 2002; He 2018), suggesting that the diversity of this species complex in East Asia remains insufficiently documented.

Members of this species complex are generally large-bodied and exhibit highly conserved external morphology, making species identification based solely on external morphology difficult (Baccetti and Capra 1978; Kang 1993; Ingrisch et al. 2006; Iorgu et al. 2016). In recent years, molecular data, particularly mitochondrial genomes, have been increasingly used to support species delimitation in Orthoptera (Cameron 2014; Pentinsaari et al. 2017; Song et al. 2019). However, mitogenomic data for the *G. gryllotalpa* species complex remain scarce.

During recent surveys in Xinjiang, northwestern China, a previously unrecognized species of *Gryllotalpa* was collected. In the present study, we describe this species as new to science based primarily on morphology, with mitochondrial DNA data provided as supplementary support. An identification key to the Chinese species of Gryllotalpidae is also provided.

## Materials and methods

### Specimen collection and morphological study

Specimens of *G. xinjiangica* Gu & Miao, sp. nov., were collected in 2022 and 2023 from two localities in the Aksu Prefecture, Xinjiang, China: Awati County (40.3883°N, 79.9939°E) and Xinhe County (41.6233°N, 82.5966°E). Live adults were anesthetized with diethyl ether, and one mid-leg was excised from each individual under a stereomicroscope. Excised tissues were immediately frozen on dry ice and used for DNA extraction. The remaining specimens were preserved in 75% ethanol and deposited in the Entomological Collection of the School of Agriculture, Ningxia University, Yinchuan, China (**NXU**).

Genitalic dissections followed Townsend (1983) and Cadena-Castañeda (2015b). The phallic complex was removed, cleared in 5% NaOH for 10–15 min, rinsed in distilled water, and preserved in glycerin.

External morphology was photographed using a Canon EOS 500D digital SLR camera. Detailed structures were imaged using a Leica M205A motorized stereomicroscope equipped with a CCD camera (Leica Microsystems, Wetzlar, Germany). Image composition and annotation were performed using Adobe Photoshop 2022 v23.4.1 (Adobe Inc., San Jose, CA, USA).

Measurements were made using ImageJ 1.50i (Schneider et al. 2012). The following parameters were recorded: **BL** (body length), **PL** (pronotum length), **PW** (pronotum width), **TL** (tegmen length), **TW** (tegmen width, including lateral field), **HFL** (hind femur length), **HTL** (hind tibia length), **SFL** (stridulatory file length), and **NST** (number of teeth on the stridulatory file). Terminology follows Tan and Kamaruddin (2016) for external morphology and Cadena-Castañeda (2015a, 2015b) for tegmen venation and genitalia. Data are presented as mean ± standard deviation (SD) and summarized separately by sex.

### Molecular analysis

Genomic DNA was extracted and sequenced by Nanjing Peisenor Biotechnology Co., Ltd. (Nanjing, China). All analyses were conducted using the invertebrate mitochondrial genetic code. Raw reads were quality-trimmed in CLC Genomics Workbench v. 7.0.4 (CLC Bio, Aarhus, Denmark), using the mitogenome of *G. unispina* (GenBank KC894752) as a reference for assembly. Genome annotation was performed in Geneious v. 8.1.3 (Kearse et al. 2012). Protein-coding genes (PCGs) were identified based on open reading frames and sequence similarity using ORF Finder (https://www.ncbi.nlm.nih.gov/orffinder/). Transfer RNA genes (tRNAs) and two ribosomal RNA genes (rRNAs) were predicted using the MITOS web server (https://usegalaxy.eu/). Circular mitogenome maps were generated using OGDRAW (Lohse et al. 2013). Base composition and codon usage were calculated in PhyloSuite v. 1.2.3 (Zhang et al. 2020).

Pairwise genetic distances based on the *cox1* barcode region were calculated under the Kimura-2-parameter (K2P) model in MEGA 7.0 (Kumar et al. 2016).

Species of Gryllotalpidae were included as the ingroup, with representatives of Myrmecophilidae as the outgroup (Table 1). Phylogenetic trees were reconstructed using maximum likelihood (ML) in IQ-TREE (Nguyen et al. 2015) with 10,000 ultrafast bootstrap replicates, and Bayesian inference (BI) in MrBayes (Ronquist et al. 2012). Partitioning schemes and substitution models were selected using PartitionFinder2 (Lanfear et al. 2017) under the corrected Akaike Information Criterion (AICc) for BI, and ModelFinder v. 2.2.0 (Kalyaanamoorthy et al. 2017) under the Bayesian Information Criterion (BIC) for ML analyses (Suppl. material 1: table SS1). Nodal support was evaluated using posterior probabilities (PP) for BI and bootstrap values (BS) for ML. Phylogenetic trees were visualized and edited in FigTree v. 1.4.4 (Rambaut 2025).

**Table 1.** Specimens included in the phylogenetic analyses.

**Species**	**Locality**	**Voucher**	**GenBank accession**	**Resource**
*Gryllotalpa gryllotalpa* (Linnaeus, 1758)	Germany	BC ZSM ORT 00043	GU706079	–
*G. africana* Palisot de Beauvois, 1820	Japan: Fukushima, Nihonmatsu	FA_9	LC619090	–
	Portugal: Beja, Mertola, Herdade de Alagaes	INV09085	OR974770	Pina et al. (2024)
*G. orientalis* Burmeister, 1838	China: Henan, Xichuan County	–	ON210982	Ma and Miao (2022)
	Republic of Korea: Gyunggi-do, Suwon City	–	NC006678	Kim et al. (2005)
*G. unispina* Saussure, 1874	China: Hebei, Shijiazhuang City	–	NC029148	Zhang et al. (2014)
*G. pluvialis* (Mjöberg, 1913)	Australia: Queensland, Brisbane	BIOUG<CAN>:AY165658	AY165658	Hebert et al. (2003)
*G. vineae* Bennet-Clark, 1970	Portugal: Setubal, Sesimbra, Pinhal da Aiana	INV02541	OR974509	Pina et al. (2024)
	Portugal: Porto, Vila do Conde, Vairao	INV04101	OR974817	Pina et al. (2024)
*G. henana* Cai & Niu, 1998	China: Henan, Xichuan County	–	NC071757	Ma and Miao (2022)
*Gryllotalpa* sp.	–	–	MK903562	Chang et al. (2020)
*G. xinjiangica* Gu & Miao, sp. nov.	China: Xinjiang, Awati County	XJM5	PV591918	This study
	China: Xinjiang, Xinhe County	XJ	PV591919	
*Myrmecophilus acervorum* (Panzer, 1799)	–	Hc4	PP896884	Kaczmarczyk-Ziemba et al. (2024)
*M. fuscus* Stalling, 2013	France: Pyrénées-Orientales, Villefranche-de-Conflent	MYR0773	MW750643	Iorgu et al. (2023)

“–” indicates that the corresponding information is unavailable.

## Results

### Taxonomic account

#### Gryllotalpa xinjiangica

Taxon classificationAnimaliaOrthopteraGryllotalpidae

Gu & Miao sp. nov.

0534FFAD-B539-55B6-84A1-7EAC128C8E04

https://zoobank.org/214F7386-1F5E-4A72-89E6-4B4E52BB7527

Figs 1, 2, 3

##### Chinese name.

新疆蝼蛄.

##### Type material.

***Holotype***: China • ♂; Xinjiang, Aksu Prefecture, Awati County, Ganquan Town, Wangou Village; 40.3883°N, 79.9939°E; alt. 1054 m; 18 Aug. 2023; Ying Miao, Ling Zhu and Jun Zhang leg.; GenBank: PV591918; NXU. ***Paratypes***: • 2 ♂, 1 ♀, same collection data as holotype • 1 ♀; Xinjiang, Aksu Prefecture, Xinhe County, Yiqi Airike Town; 41.6233°N, 82.5966°E; alt. 1023 m; 30 Jul. 2022; Xin-Pu Wang, Yan Ma and Yu-Chen Zhao leg.; GenBank: PV591919; NXU.

##### Diagnosis.

*Gryllotalpa xinjiangica* sp. nov. is a large mole cricket (body length > 40 mm) characterized by the following combination of characters: body generally greyish brown with yellowish ocelli; pronotum ovoid, bearing a distinct median Y-shaped longitudinal stripe; forelegs fossorial, with four tibial dactyls; hind tibiae lacking inner subapical spurs; R vein of the tegmina branching into RA and RP; male stridulatory file with 69–83 teeth (22–26 teeth mm^−1^); hind wings reaching the abdominal apex; epiphallus hourglass-shaped; apex of the median prolongation fan-shaped in dorsal view, approximately as wide as or slightly wider than the base of the median prolongation; transverse sclerite L-shaped in caudal view and V-shaped in dorsal view.

##### Description.

**Figure 1.**
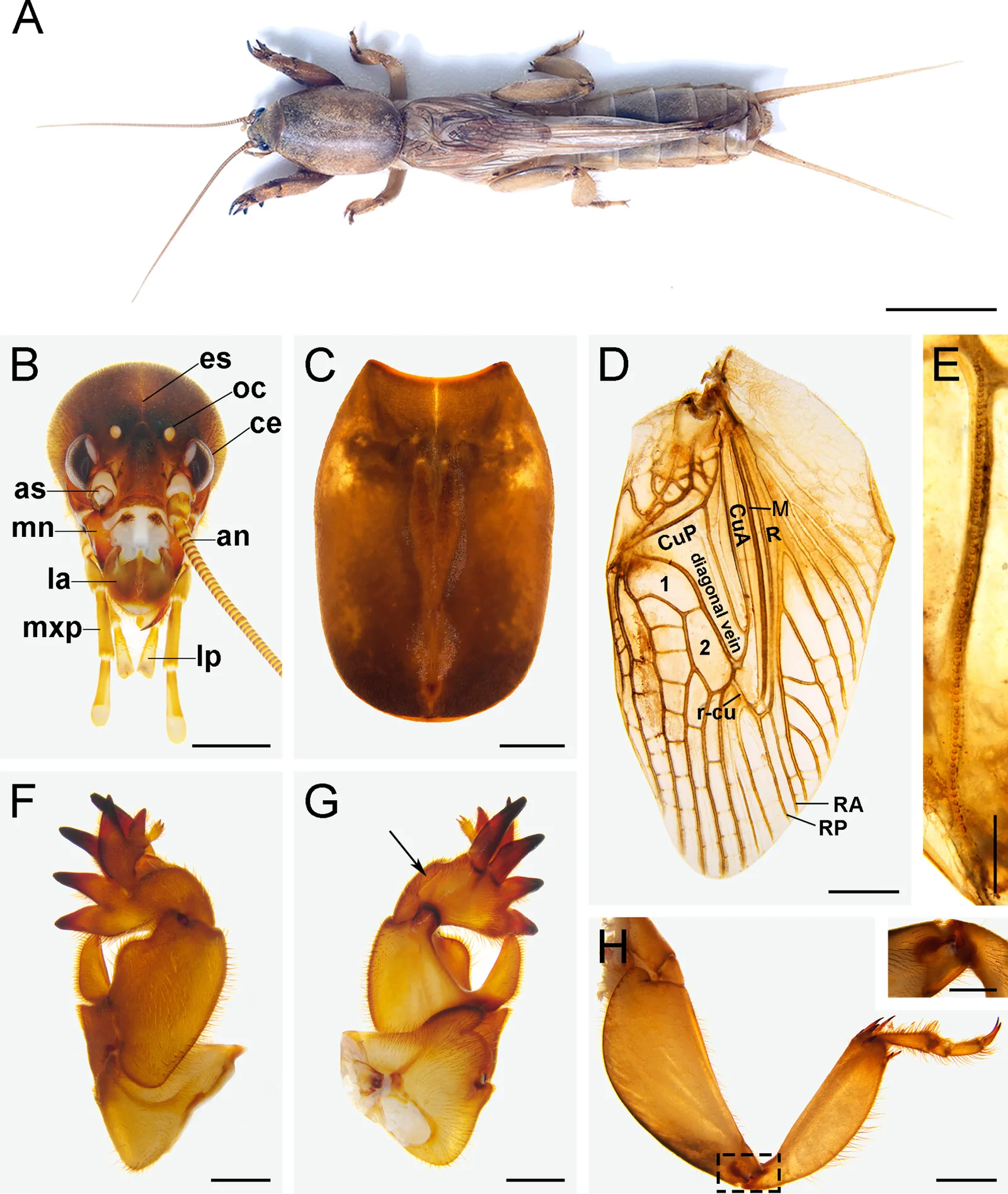
Holotype of *Gryllotalpa xinjiangica* sp. nov. (male). **A**. Adult, dorsal view; **B**. Head, dorsal view; **C**. Pronotum, dorsal view; **D**. Tegmen showing cells 1 (1) and 2 (2), dorsal view; **E**. Stridulatory file on CuP; **F**. Foreleg, external view; **G**. Foreleg, internal view; arrow indicating the tympanic organ; **H**. Hind leg, external view; inset showing a close-up of the semilunar process. Abbreviations: an = antenna, as = antennal socket, ce = compound eye, es = ecdysial suture, la = labrum, lp = labial palpus, mn = mandible, mxp = maxillary palpus, oc = ocellus, CuA = anterior cubitus, CuP = posterior cubitus, M = media, R = radius, RA = anterior radius, RP = posterior radius, r-cu = radio-cubital crossvein. Scale bars: 1 cm (**A**); 2 mm (**B–D, F–H**); 500 µm (**E** and inset of **H**).

**Figure 2.**
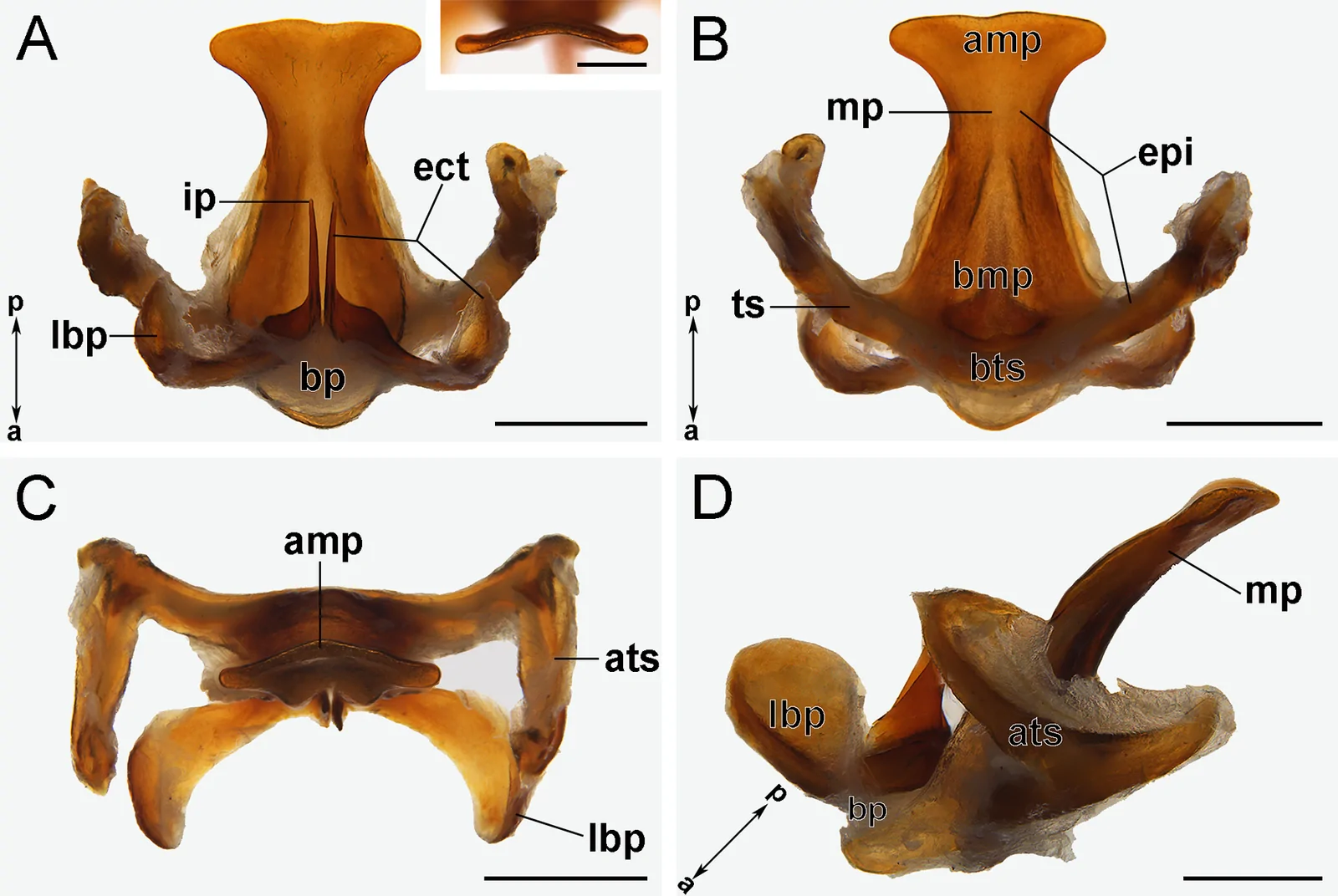
Male phallic complex of *Gryllotalpa xinjiangica* sp. nov. **A**. Ventral view; inset showing the apex of the median prolongation; **B**. Dorsal view; **C**. Caudal view; **D**. Lateral view. Arrows indicate the position of the phallic complex; a, anterior; p, posterior. Abbreviations: amp = apex of the median prolongation, ats = apex of the transverse sclerite, bmp = base of the median prolongation, bp = basal plate, bts = base of the transverse sclerite, ect = ectophallus, epi = epiphallus, mp = median prolongation, lbp = latero-basal plate, ip = internal prolongations, ts = transverse sclerite. Scale bars: 1 mm (**A–D**); 500 µm (inset of **A**).

**Male** (holotype; Figs 1, 2). Body large, generally greyish brown (Fig. 1A). ***Head*** (Fig. 1B): vertex brown; ocelli small, ovoid, orange; compound eyes prominent, rounded; antennae yellowish brown, approximately twice as long as the pronotum. ***Thorax*** (Fig. 1C, F–H): pronotum (Fig. 1C) ovoid, 1.5× as long as wide, bearing a distinct median Y-shaped longitudinal stripe; anterior margin emarginate; posterior margin rounded. Fore coxa (Fig. 1F, G) robust, densely covered with long setae, with blunt processes along the anterior margin; fore femur subrectangular, with a distinct median protuberance on the ventral margin; fore tibial dactyls moderately long, ventral dactyl straight, dorsal dactyls slightly curved; inner tympanum (Fig. 1G) slit-shaped. Hind tibia (Fig. 1H) lacking inner subapical spurs; apex with four medial and three lateral spurs. ***Wings*** (Fig. 1A, D, E): tegmina (Fig. 1D) 1.78× as long as wide, overlapping; venation symmetrical. R vein branching into RA and RP. M vein distinct, its distal portion bifurcating into two branches, the anterior branch joining RA and the posterior branch joining the r-cu crossvein. Harp enclosed by CuA, CuP, and the diagonal vein, inverted triangular in shape, tapering distally; cell 1 (c1) ~ 1.08× as large as cell 2 (c2). Stridulatory file (Fig. 1E) sinuate, with 69 teeth (22 teeth mm^−1^). Hind wings fully developed, basal one-third concealed beneath the tegmina (Fig. 1A). ***Abdomen*** (Fig. 1A): slender and elongate, abdominal apex cylindrical; dorsum greyish brown. ***Phallic complex*** (Fig. 2): ectophallus with paired sclerotized parameres, each bearing an acute internal prolongation (Fig. 2A); latero-basal plate (Fig. 2A, C, D) gently curved upward along ventral margin; basal plate ovoid in ventral view (Fig. 2A), subquadrate in lateral view (Fig. 2D). Epiphallus (Fig. 2B) hourglass-shaped; median prolongation of moderate length; apex of the median prolongation (Fig. 2C) fan-shaped in dorsal view, as wide as or slightly wider than its base; apical margin (Fig. 2C, inset) distinctly emarginate in caudal view. Transverse sclerite (Fig. 2B) narrow, V-shaped in dorsal view and L-shaped in caudal view; apex of the transverse sclerite (Fig. 2C, D) bifurcated, forming a short dorsal ramus and a long ventral ramus, the latter apically hooked.

**Figure 3.**
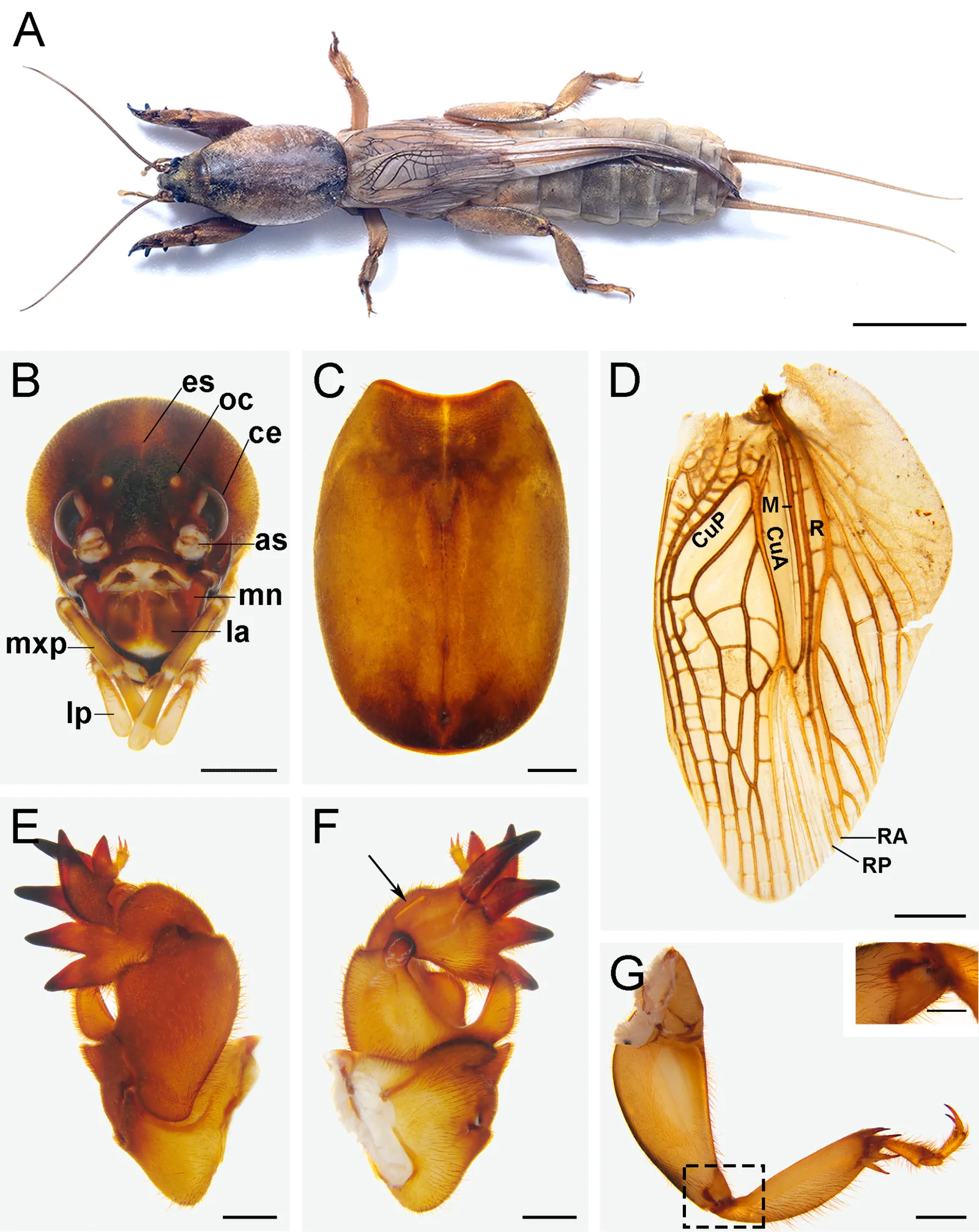
Paratype of *Gryllotalpa xinjiangica* sp. nov. (female). **A**. Adult, dorsal view; **B**. Head, dorsal view; **C**. Pronotum, dorsal view; **D**. Tegmen, dorsal view; **E**. Foreleg, external view; **F**. Foreleg, internal view; arrow indicating the tympanic organ; **G**. Hind leg, internal view; inset showing a close-up of the semilunar process. Abbreviations: as = antennal socket, ce = compound eye, es = ecdysial suture, la = labrum, lp = labial palpus, mn = mandible, mxp = maxillary palpus, oc = ocellus, CuA = anterior cubitus, CuP = posterior cubitus, M = media, R = radius, RA = anterior radius, RP = posterior radius. Scale bars: 1 cm (**A**), 2 mm (**B–G**); 500 µm (inset of **G**).

**Female** (Fig. 3). Similar to male, differing mainly in size and several secondary characters. Body larger than in the male; coloration similar (Fig. 3A). ***Head*** (Fig. 3B): vertex brown, broader than in male; ocelli and compound eyes similar to those of the male; antennae brown, ~ 1.5× as long as the pronotum. ***Thorax*** (Fig. 3C, E–G): pronotum (Fig. 3C) similar in shape to that of the male, slightly larger than that of the male; median Y-shaped stripe indistinct. Forelegs (Fig. 3E, F) similar to those of the male but more robust. Hind legs (Fig. 3G) similar, with femur and tibia slightly broader. ***Wings*** (Fig. 3A, D): tegmina (Fig. 3D) similar in size to those of the male, lacking an inverted-triangular harp; R vein branching as in the male; hind wings fully developed, surpassing the abdominal apex (Fig. 3A). ***Abdomen*** (Fig. 3A): more robust than in the male, not elongate; terminally cylindrical; dorsum light brown.

##### Measurements.

(means ± SD; Suppl. material 1: table SS2) ***Male*** (*n* = 3): BL 4.64 ± 0.09 cm; PL 1.07 ± 0.06 cm; PW 0.71 ± 0.02 cm; TL 1.44 ± 0.11 cm; TW 0.81 ± 0.04 cm; HFL 0.83 ± 0.02 cm; HTL 0.65 ± 0.01 cm; SFL 0.32 ± 0.01 cm; NST 77.33 ± 7.37. ***Female*** (*n* = 2): BL 5.23 ± 0.04 cm; PL 1.39 ± 0.17 cm; PW 0.98 ± 0.08 cm; TL 1.55 ± 0.02 cm; TW 0.87 ± 0.04 cm; HFL 1.09 ± 0.01 cm; HTL 0.72 ± 0.01 cm.

##### Etymology.

The specific epithet refers to Xinjiang, the type locality of the species.

##### Distribution.

Known only from Xinjiang Uygur Autonomous Region, northwestern China.

##### Remarks.

*Gryllotalpa xinjiangica* sp. nov. belongs to the *G. gryllotalpa* species complex. It most closely resembles *G. unispina*, but differs in several diagnostic features. The new species is generally greyish brown, whereas *G. unispina* is predominantly yellowish brown (Saussure 1877). In males, the stridulatory file bears fewer and more loosely arranged teeth (69–83 teeth; 22–26 teeth mm^−1^), compared with 94–117 teeth (~ 41 teeth mm^−1^) in *G. unispina* (Hu et al. 2026). The hind wings reach the abdominal apex, whereas those of *G. unispina* distinctly surpass it (Saussure 1877). The male genitalia provide additional distinguishing characters. In *G. xinjiangica* sp. nov., the apex of the median prolongation is fan-shaped in dorsal view and is as wide as or slightly wider than its base, whereas in *G. unispina* it is shovel-shaped and noticeably narrower than the base of the median prolongation (Iorgu et al. 2016). In caudal view, the apical margin of the median prolongation is distinctly emarginate in the new species but only shallowly emarginate in *G. unispina*. The transverse sclerite is L-shaped in caudal view in both species but differs in dorsal view, appearing V-shaped in *G. xinjiangica* sp. nov. and broadly U-shaped in *G. unispina*. These differences were consistent across all examined specimens and, together with the other diagnostic characters, support the recognition of *G. xinjiangica* sp. nov. as a distinct species.

### Key to males of species of *Gryllotalpa* from China

The following key is based on adult morphological characters and includes all currently known species of *Gryllotalpa* from China. This key is modified from Ma et al. (2008), Ma and Zhang (2011), Tan (2016), and Yang (1995). *Gryllotalpa chinensis* is excluded due to insufficient diagnostic information and the absence of the type specimen.

**Table:** 

1	Body length greater than 40 mm	**2**
–	Body length less than 40 mm	**3**
2	Apex of median prolongation shovel-shaped in dorsal view, distinctly narrower than its base; apical margin shallowly emarginate in caudal view	***G. unispina* Saussure, 1874**
–	Apex of median prolongation fan-shaped in dorsal view, as wide as or slightly wider than its base; apical margin distinctly emarginate in caudal view	***G. xinjiangica* Gu & Miao, sp. nov**.
3	Tegmina > 10 mm; hind wings fully developed, extending beyond the apex of the hind femur	***G. orientalis* Burmeister, 1838**
–	Tegmina < 10 mm; hind wings vestigial, concealed beneath the tegmina	**4**
4	Tegmen not reaching the posterior margin of the third abdominal segment	**5**
–	Tegmen reaching the posterior margin of the third abdominal segment	**6**
5	Tegmina not reaching posterior margin of the second abdominal segment; tympanal opening of the fore tibiae ~ 0.14× the length of the ventral margin	***G. jinxiuensis* You & Li, 1990**
–	Tegmina reaching posterior margin of the second abdominal segment; tympanal opening ~ 0.12× the length of the ventral margin	***G. formosana* Shiraki, 1930**
6	Hind wing extending slightly beyond the apex of the tegmina	***G. dentista* Yang, 1995**
–	Hind wing not reaching the apex of the tegmina	**7**
7	Transverse sclerite distinctly T-shaped in caudal view	**8**
–	Transverse sclerite L-shaped in caudal view	**11**
8	Body length < 22 mm; tegmina < 7.5 mm; lateral tip of transverse sclerite acute and hooked	***G. mabiana* Ma, Xu & Takeda, 2008**
–	Body length > 24 mm; tegmina > 7.5 mm; lateral tip of transverse sclerite neither acute nor hooked	**9**
9	Tegminal cells c1 and c2 closed; transverse sclerite robust; parameres rounded	***G. wudangensis* Li, Ma & Xu, 2007**
–	Tegminal cells c1 and c2 open distally	**10**
10	Epiphallus scoop-like, nearly flat basally; apex slightly upcurved	***G. henana* Cai & Niu, 1998**
–	Epiphallus widened; lower portion well-curved	***G. breviabdominis* Ma & Zhang, 2011**
11	Tegmina subcircular when flattened	**12**
–	Tegmina inverted triangular when flattened	***G. chrysea* Ma, Yuan & Wang, 2024**
12	Body length ~ 27 mm; tegmina short, covering the first three abdominal segments; only cell c1 present	***G. cycloptera* Ma & Zhang, 2011**
–	Body length ~ 24 mm; tegmina longer; cells c1 and c2 present	***G. choui* Ma & Zhang, 2010**

### Mitogenomic characteristics

**Figure 4.**
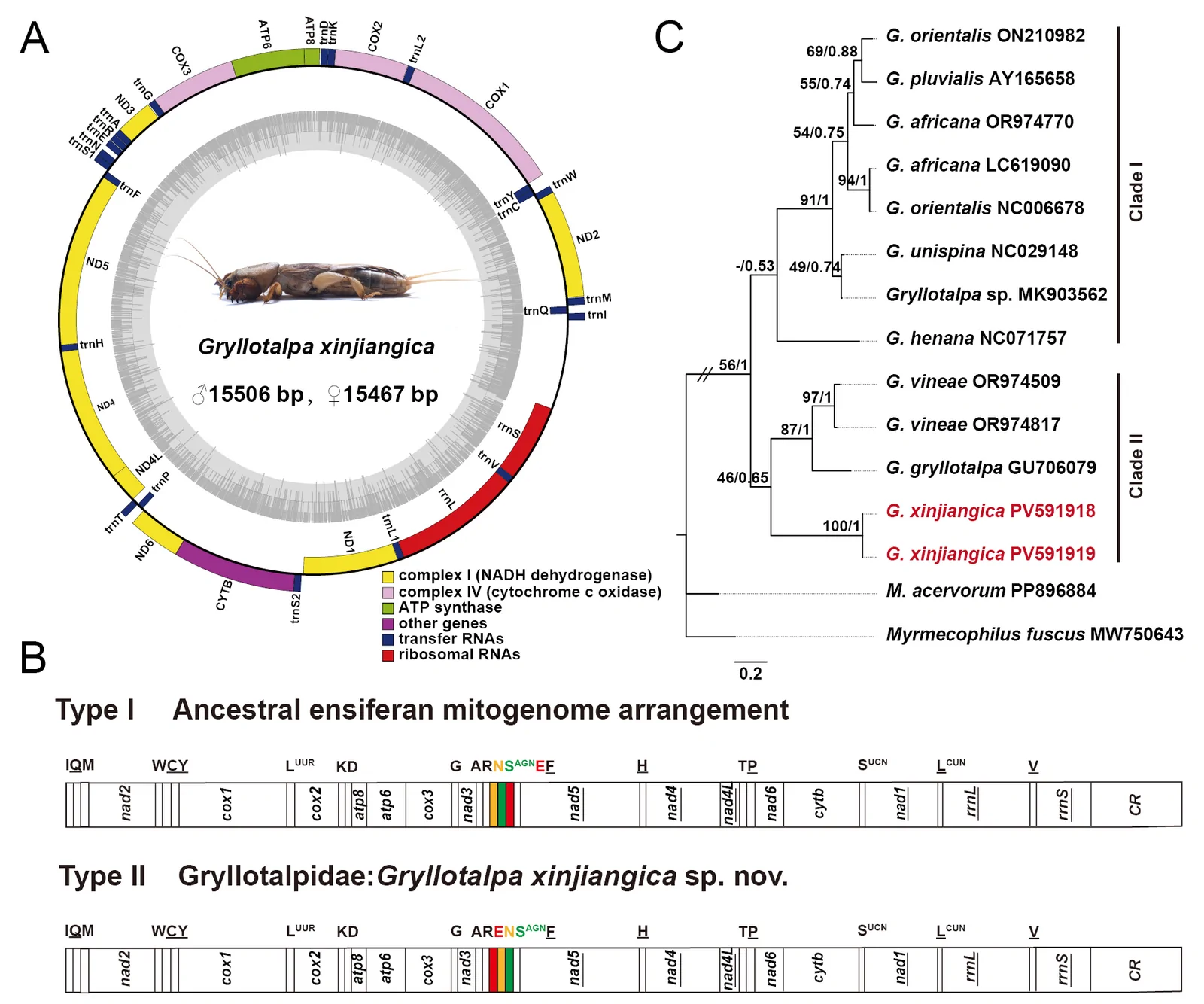
Mitochondrial genome structure, gene arrangement, and phylogenetic position of *Gryllotalpa xinjiangica* sp. nov. **A**. Circular map of the mitochondrial genome. Genes encoded on the J-strand are shown on the outer circle, and those on the N-strand on the inner circle. The grey ring represents nucleotide composition, with darker and lighter regions indicating GC and AT content, respectively; **B**. Mitochondrial gene arrangements in ancestral Ensifera (Type I) and *G. xinjiangica* sp. nov. (Type II); **C**. Phylogenetic tree inferred from the *cox1* dataset using Bayesian inference (BI). ML bootstrap support values (BS) and Bayesian posterior probabilities (PP) are indicated at internal nodes. “–” indicates that bootstrap support is not shown for that node.

The complete mitochondrial genomes of the male and female *G. xinjiangica* sp. nov. (GenBank accession numbers PV591918 and PV591919) are circular DNA molecules with lengths of 15,506 bp and 15,467 bp, respectively. Each mitogenome comprises the typical set of 37 genes (13 PCGs, 22 tRNAs, and two rRNAs) and an AT-rich control region (Fig. 4A). Gene content and organization are generally consistent with those of other gryllotalpid mitogenomes (Table 2).

**Table 2.** Mitogenomic organization of *Gryllotalpa xinjiangica* sp. nov.

**Gene**	**Position**		**Size**	**IGN**	**Codon**		**Direction**
	**From**	**To**			**Start**	**Stop**	
*trnI*	1/1	67/67	67/67	–/–			J/J
*trnQ*	64/65	137/133	74/69	−4/−3			N/N
*trnM*	143/140	212/208	70/69	5/6			J/J
*nad2*	219/215	1211/1207	993/993	6/6	ATA/ATA	TAA/TAA	J/J
*trnW*	1209/1206	1277/1273	69/68	−3/−2			J/J
*trnC*	1269/1266	1331/1327	63/62	−9/−8			N/N
*trnY*	1331/1327	1394/1390	64/64	−1/−1			N/N
*cox1*	1397/1393	2930/2926	1534/1534	2/2	ATG/ATG	T/T	J/J
*trnL2*	2930/2927	2995/2991	66/65	−1/–			J/J
*cox2*	2997/2993	3675/3671	679/679	1/1	ATG/ATG	T/T	J/J
*trnK*	3675/3672	3742/3739	68/68	−1/–			J/J
*trnD*	3742/3739	3807/3803	66/65	−1/−1			J/J
*atp8*	3817/3813	3963/3959	147/147	9/9	ATA/ATA	TAA/TAA	J/J
*atp6*	3960/3956	4634/4630	675/675	−4/−4	ATA/ATA	TAA/TAA	J/J
*cox3*	4634/4630	5417/5413	784/784	−1/−1	ATG/ATG	T/T	J/J
*trnG*	5417/5414	5481/5476	65/63	−1/–			J/J
*nad3*	5490/5486	5834/5830	345/345	8/9	TTA/TTA	TAG/TAG	J/J
*trnA*	5833/5829	5895/5890	63/62	−2/−2			J/J
*trnR*	5897/5893	5958/5953	62/61	1/2			J/J
*trnE*	5963/5959	6026/6021	64/63	4/5			J/J
*trnN*	6070/6067	6138/6132	69/66	43/45			J/J
*trnS1*	6138/6134	6199/6194	62/61	−1/1			J/J
*trnF*	6211/6207	6277/6272	67/66	11/12			N/N
*nad5*	6277/6273	7996/7992	1720/1720	−1/–	ATG/ATG	T/T	N/N
*trnH*	7997/7993	8060/8055	64/63	–/–			N/N
*nad4*	8060/8056	9392/9388	1333/1333	−1/–	ATG/ATG	T/T	N/N
*nad4L*	9386/9382	9682/9678	297/297	−7/−7	ATG/ATG	TAA/TAA	N/N
*trnT*	9685/9681	9749/9744	65/64	2/2			J/J
*trnP*	9749/9745	9811/9806	63/62	−1/–			N/N
*nad6*	9831/9827	10316/10312	486/486	19/20	ATA/ATA	TAA/TAA	J/J
*cytb*	10316/10312	11449/11445	1134/1134	−1/−1	ATG/ATG	TAA/TAA	J/J
*trnS2*	11448/11444	11514/11509	67/66	−2/−2			J/J
*nad1*	11531/11527	12465/12461	935/935	16/17	ATA/ATA	TA/TA	N/N
*trnL1*	12467/12463	12531/12526	65/64	1/1			N/N
*rrnL*	12531/12527	13794/13754	1264/1228	–/–			N/N
*trnV*	13795/13755	13863/13823	69/69	–/–			N/N
*rrnS*	13864/13824	14630/14591	767/768	–/–			N/N
CR	14631/14592	15506/15467	876/876	–/–			

Data are presented separately for the male and female mitogenomes. CR, control region; IGN, intergenic nucleotides. Negative values indicate overlaps between adjacent genes. “–” indicates that no intergenic nucleotides are present.

The overall AT content is 71.00% in the male and 71.10% in the female, indicating a strong AT bias (Suppl. material 1: table S3). Most PCGs initiate with the standard ATN codons, except *nad3*, which starts with TTA (Table 2). Typical cloverleaf secondary structures are present in all tRNAs except *trnS1*, in which the dihydrouridine (DHU) arm is reduced (Suppl. material 2: figs S1, S2).

A rearrangement of the mitochondrial tRNA gene cluster (*trnE-trnN-trnS1*) was detected, differing from the putative ancestral insect gene order (*trnN-trnS1-trnE*) (Fig. 4B).

### Molecular phylogeny

Phylogenetic analyses of the *cox1* dataset using Bayesian inference (BI) and maximum likelihood (ML) yielded congruent topologies (Fig. 4C). The sampled *Gryllotalpa* species were recovered in two major clades (I and II), with strong Bayesian support (PP = 1) but only moderate ML bootstrap support (BS = 56). *Gryllotalpa xinjiangica* sp. nov. was resolved as the sister lineage to the clade comprising *G. vineae* and *G. gryllotalpa*, although support for this relationship was moderate (PP = 0.65, BS = 46).

Pairwise genetic distances (K2P) based on *cox1* between *G. xinjiangica* sp. nov. and its congeners ranged from 0.21–0.25, values comparable to or exceeding those of reported interspecific divergence within *Gryllotalpa* (0.02–0.26; Suppl. material 1: table S4). These molecular results are consistent with the morphological evidence and support the recognition of *G. xinjiangica* sp. nov. as a distinct species.

## Discussion

Species delimitation within *Gryllotalpa* has long been recognized as challenging because many species exhibit highly conserved external morphology. Taxonomic revisions of European members of the *G. gryllotalpa* species complex, particularly in Italy, the Balkan Peninsula, and adjacent regions, have shown that reliable species identification depends primarily on the morphology of the male genitalia (Ingrisch et al. 2006; Iorgu et al. 2016; Bogdanović et al. 2017) and karyotypic evidence in some cases (Ortiz 1958; Baccetti and Capra 1978; Baccetti 1991, 1992). These studies have also demonstrated that morphologically similar populations may represent distinct species despite their highly similar external morphology.

Morphological evidence indicates that *G. xinjiangica* sp. nov. belongs to the *G. gryllotalpa* species complex. The new species shares with members of this complex several characteristic features, including a relatively large body size, well-developed wings, a characteristic pattern of tegminal venation, and an hourglass-shaped epiphallus of the male genitalia. These characters have long been regarded as diagnostic features of this complex (Bennet-Clark 1970; Baccetti and Capra 1978; Baccetti 1992; Gorochov 1993; Kang 1993; Broza et al. 1998; Cai and Niu 2002; Ingrisch et al. 2006; Iorgu et al. 2016; Bogdanović et al. 2017).

Although body size alone is not suitable for species delimitation, it provides a useful preliminary criterion for restricting comparisons among morphologically similar species (Tan 2016). Most described *Gryllotalpa* species from Africa, India, and Southeast Asia are distinctly smaller than *G. xinjiangica* sp. nov. (Townsend 1983; Tan 2016; Simeu-Noutchom et al. 2020, 2021). Consequently, detailed comparisons were focused primarily on members of the *G. gryllotalpa* species complex and the few other large-bodied Southeast Asian species, namely *G. fraser* Tan & Kamaruddin, 2013, *G. hirsuta* Burmeister, 1838, and *G. nymphicus* Tan, 2012.

The large-bodied Malaysian species (*G. fraser*, *G. hirsuta* and *G. nymphicus*) are readily distinguished from *G. xinjiangica* sp. nov. by their strongly abbreviated fore and hind wings (Tan 2012; Tan and Kamaruddin 2013). They also differ markedly in the configuration of the male genitalia.

Comparison of the tegminal venation reveals several consistent differences among members of the *G. gryllotalpa* species complex. In *G. xinjiangica* sp. nov., cell c1 is slightly larger than c2, and the distinct M vein bifurcates distally into two branches, the anterior joining RA and the posterior joining the r-cu crossvein. By contrast, several members of the complex, including *G. cossyrensis*, *G. marismortui*, *G. quindecim*, *G. septemdecimchromosomica*, *G. tali*, and *G. viginti*, differ in the relative proportions of cells c1 and c2 and/or in the course of the median vein (Baccetti and Capra 1978; Broza et al. 1998). The male stridulatory apparatus also provides useful diagnostic evidence. For example, *G. vineae* possesses only 42–50 teeth, whereas *G. unispina* exhibits a denser stridulatory file with substantially more teeth per millimetre (Bennet-Clark 1970; Hu et al. 2026).

The morphology of the median prolongation provides one of the most informative diagnostic characters within the *G. gryllotalpa* species complex. Species such as *G. cossyrensis*, *G. isfahan*, *G. krimbasi*, *G. octodecim*, *G. quindecim*, *G. sedecim*, *G. septemdecimchromosomica*, *G. stepposa*, *G. viginti*, and *G. vineae* possess a relatively narrow, trough-shaped apex that is approximately half as wide as the base of the median prolongation, with nearly parallel or only slightly divergent lateral margins (Baccetti and Capra 1978; Zhantiev 1991; Ingrisch et al. 2006; Iorgu et al. 2016; Bogdanović et al. 2017). In *G. gryllotalpa*, the apex remains trough-shaped but is shorter and broader, with distinctly divergent lateral margins, although it is still narrower than the base (Baccetti and Capra 1978; Zhantiev 1991; Ingrisch et al. 2006; Iorgu et al. 2016). *Gryllotalpa unispina* exhibits a shovel-shaped apex that is broader than in the preceding species but still distinctly narrower than the base of the median prolongation (Cai and Niu 2002; Iorgu et al. 2016). In contrast, *G. xinjiangica* sp. nov. is characterized by a distinctly expanded fan-shaped apex, which is as wide as or slightly wider than the base of the median prolongation. The transverse sclerite also differs markedly among species. In *G. xinjiangica* sp. nov. it is V-shaped in dorsal view, whereas it is broadly U-shaped in *G. isfahan*, *G. stepposa*, *G. unispina*, and several other members of the complex (Baccetti 1992; Cai and Niu 2002; Ingrisch et al. 2006; Iorgu et al. 2016; Bogdanović et al. 2017).

No single morphological character is sufficient to distinguish *G. xinjiangica* sp. nov. from all congeners. However, no previously described species combines the absence of inner subapical spurs on the hind tibiae, the characteristic tegminal venation, the relatively sparse stridulatory file, and the unique configuration of the median prolongation and transverse sclerite. Comparison with the original descriptions and subsequent taxonomic revisions indicates that the Xinjiang population does not correspond to any previously described species of the *G. gryllotalpa* species complex or other morphologically similar *Gryllotalpa* species, supporting its recognition as a distinct species.

The molecular evidence is consistent with the morphological observations and supports the recognition of *G. xinjiangica* sp. nov. as a distinct species. Phylogenetic analyses based on the *cox1* dataset consistently recovered the new species as an independent lineage within *Gryllotalpa*. Although support for several deeper nodes was only moderate or weak, the placement of *G. xinjiangica* sp. nov. as a distinct lineage remained stable across analytical methods and agrees with the morphological evidence. These results indicate that the molecular data provide independent support for the morphological species hypothesis, while additional molecular markers and broader taxon sampling will be valuable for resolving relationships within the genus.

The mitochondrial genome of *G. xinjiangica* sp. nov. is generally conserved in gene content and overall organization relative to other gryllotalpid species. However, a rearrangement of the tRNA gene cluster (*trnE-trnN-trnS1*) was detected, resulting in an arrangement that differs from the putative ancestral insect gene order (Cameron 2014). Similar rearrangements involving this tRNA cluster have also been reported in other members of Gryllidea (Ma and Li 2018; Ma et al. 2019; Yu et al. 2024), suggesting that this region may represent a hotspot for mitochondrial genome rearrangement within the superfamily.

Although mitochondrial genome organization alone cannot serve as diagnostic evidence for species delimitation, the newly generated mitogenome provides valuable comparative data for Gryllotalpidae, a family for which genomic resources remain limited. The concordance among diagnostic morphology, mitochondrial phylogeny and mitogenome organization provides complementary evidence supporting the recognition of *G. xinjiangica* sp. nov. as a distinct species and provides a useful foundation for future studies on the taxonomy and evolutionary history of mole crickets.

## Supplementary Material

XML Treatment for Gryllotalpa xinjiangica

## Additional information

### Artificial Intelligence (AI) use

ChatGPT was used solely for language editing, including improving the English grammar and syntax of this manuscript. The authors are fully responsible for the content of the manuscript.

### Author ORCIDs

Y.-R. Gu https://orcid.org/0009-0009-2698-3344

X.-L. Deng https://orcid.org/0009-0005-4560-1250

L. Zhu https://orcid.org/0009-0009-6351-8986

Y. Ma https://orcid.org/0009-0000-5389-3293

Y. Miao https://orcid.org/0000-0001-6861-0129

### Data availability

The newly generated mitochondrial genome sequences have been deposited in GenBank under accession numbers PV591918–PV591919. The nomenclatural act has been registered in ZooBank under the LSID: https://zoobank.org/A40A278D-130E-44A8-93F1-F35BA3942AC4. All additional data, including phylogenetic trees, sequence alignments, and morphological measurements, are available in the main text, figures, tables, and Supplementary Information. Additional files are available from the corresponding author upon reasonable request.

## Supplementary materials

Supplementary material 1 Supplementary tables Data type xls Explanation note **table SS1**. Partitioning schemes and substitution models. **table SS2**. Morphological measurements. **table S3**. Nucleotide composition of the mitogenomes. **table S4**. Matrix of evolutionary distances. https://binary.pensoft.net/file/1762751This dataset is made available under the Open Database License (http://opendatacommons.org/licenses/odbl/1.0/). The Open Database License (ODbL) is a license agreement intended to allow users to freely share, modify, and use this Dataset while maintaining this same freedom for others, provided that the original source and author(s) are credited. Ya-Rong Gu, Xin-Lan Deng, Ling Zhu, Yan Ma, Ying Miao

Supplementary material 2 Predicted secondary structures of mitochondrial tRNA genes Data type doc https://binary.pensoft.net/file/1762752This dataset is made available under the Open Database License (http://opendatacommons.org/licenses/odbl/1.0/). The Open Database License (ODbL) is a license agreement intended to allow users to freely share, modify, and use this Dataset while maintaining this same freedom for others, provided that the original source and author(s) are credited. Ya-Rong Gu, Xin-Lan Deng, Ling Zhu, Yan Ma, Ying Miao
