# Supplementary material for: A new species of Gryllotalpa (Orthoptera, Gryllotalpidae) from northwestern China, with notes on its mitochondrial genome
Source: Zookeys. 2026 Sep 2;1291:75–92. doi: 10.3897/zookeys.1291.203191 (PMC13559519; doi:10.3897/zookeys.1291.203191)
Supplement: Supplementary file 1 [file zookeys-1291-075_article-203191__-s002.doc]

**Supplementary Material Information**

**File content: Supplementary Figures S1–S2**

**Article title:** A new species of *Gryllotalpa* (Orthoptera, Gryllotalpidae) from northwestern China, with notes on its mitochondrial genome

**Journal:** Zookeys

**Authors:** YA-RONG GU, XIN-LAN DENG, LING ZHU, YAN MA, YING MIAO

**Affiliation of corresponding author:** *School of Agriculture, Ningxia University, Yinchuan 750021, China*

**Corresponding author:** Ying Miao ([miaoy@nxu.edu.cn](mailto:miaoy@nxu.edu.cn))

**Figure S1** Predicted secondary structures of mitochondrial tRNA genes in male *Gryllotalpa xinjiangica* sp. nov.

**Figure S2** Predicted secondary structures of mitochondrial tRNA genes in female *Gryllotalpa xinjiangica* sp. nov.
